# Nutritional Management in Intensive Care: From Nutritional Support to Personalized Nutritional Therapy

**DOI:** 10.3390/nu18142315

**Published:** 2026-07-15

**Authors:** Jae Gil Lee

**Affiliations:** Department of Surgery, Ewha Womans University Mokdong Hospital, 1071 Anyangcheon-ro, Yangcheon-gu, Seoul 07985, Republic of Korea; jakii71@gmail.com

## 1. Introduction

Nutrition plays a fundamental role in the management of critically ill patients and is increasingly recognized as a cornerstone of intensive care therapy. Traditionally regarded as supportive care, nutritional therapy is now considered an active intervention that influences metabolism, immune function, skeletal muscle preservation, and recovery from critical illness [1,2,3,4]. Malnutrition and rapid muscle wasting frequently occur during intensive care unit (ICU) admission and are associated with prolonged mechanical ventilation, increased complications, delayed recovery, and poor long-term outcomes [1,2]. Consequently, nutritional therapy has evolved beyond simply providing calories and protein to encompass preservation of physical function and improvement of long-term quality of life.

Recent advances in critical care nutrition have resulted in important paradigm shifts. Rather than maximizing caloric delivery during the acute phase of illness, current evidence supports avoiding both underfeeding and overfeeding while tailoring nutritional interventions according to disease phase and metabolic status [1,2,3,4]. Similarly, protein provision is no longer regarded as a “more is better” strategy but rather as an individualized intervention targeting approximately 1.2–1.3 g/kg/day in most critically ill patients [1,2,3,4]. Nutritional adequacy remains an important goal; however, contemporary nutritional therapy increasingly focuses on delivering the right nutrients, to the right patient, at the right time. In parallel, concepts such as personalized nutrition, metabolic monitoring, multidisciplinary care, and recovery-oriented nutritional support have gained increasing attention [5,6].

In this context, the Special Issue “Nutritional Management in Intensive Care” was launched to provide an updated overview of current evidence and emerging concepts in ICU nutrition. The Special Issue includes nine articles comprising one systematic review and meta-analysis, five narrative reviews, and three original research articles. Collectively, these studies address key aspects of contemporary critical care nutrition, including nutritional delivery strategies, assessment and monitoring, muscle preservation, metabolic management, multidisciplinary care, and personalized nutritional therapy.

## 2. Optimizing Nutritional Delivery

The route and timing of nutritional support remain central issues in critically ill patients. Baik et al. (Contribution 1) conducted a systematic review and meta-analysis comparing early enteral nutrition (EEN) with early parenteral nutrition (EPN) in critically ill patients. Although EEN did not demonstrate a mortality benefit, it was associated with reduced bloodstream infections and shorter ICU and hospital lengths of stay. These findings reinforce current guideline recommendations favoring enteral nutrition whenever feasible while emphasizing individualized nutritional decision-making based on gastrointestinal function.

Nutritional therapy should not end with successful enteral feeding initiation. Vinci et al. (Contribution 2) addressed the transition from enteral to oral nutrition in ICU and post-ICU patients. Their scoping review highlighted the importance of structured transition strategies and ongoing nutritional monitoring during recovery. Premature discontinuation of enteral feeding before sufficient oral intake may lead to nutritional deterioration, emphasizing that nutritional management should continue throughout rehabilitation. Together, these contributions emphasize that nutritional adequacy should be viewed as a continuum extending from the acute phase of critical illness to post-ICU recovery.

## 3. Protein Provision and Functional Recovery

One of the most important shifts in critical care nutrition is the growing recognition that preservation of muscle mass and physical function should be considered key therapeutic goals. ICU-acquired weakness (ICUAW) is strongly associated with prolonged ICU stay, delayed rehabilitation, and long-term disability.

Formenti et al. (Contribution 3) reviewed the combined effects of nutritional therapy and early mobilization on ICUAW. Their work highlights the synergistic relationship between nutrition and rehabilitation and supports multidisciplinary interventions to preserve muscle mass and improve functional outcomes.

Similarly, Scarcella et al. (Contribution 4) investigated the impact of protein supplementation on skeletal muscle architecture in critically ill patients. Their pilot study suggested that whey protein supplementation and specific amino acid formulations, including β-hydroxy β-methylbutyrate (HMB), may contribute to maintaining muscle integrity. Although larger studies are needed, these findings reflect a broader shift from calorie-focused feeding toward nutritional strategies aimed at preserving muscle mass and physical function.

Taken together, these studies reflect the broader transition from calorie-focused nutritional support toward muscle-targeted and recovery-oriented nutritional therapy.

## 4. Personalized Nutrition and Nutritional Assessment

Personalized nutrition and nutritional assessment represent another major direction in contemporary critical care nutrition. Stoian et al. (Contribution 5) reviewed future perspectives on personalized nutritional strategies in ICU patients. The authors discussed the potential role of artificial intelligence, predictive analytics, metabolic monitoring, and precision medicine approaches in guiding individualized nutritional prescriptions. As technologies such as indirect calorimetry, body composition assessment, and biomarker-guided interventions become increasingly available, personalized nutritional therapy may become more feasible in routine clinical practice.

These developments suggest that future nutritional strategies may increasingly rely on real-time metabolic assessment and adaptive therapeutic interventions.

## 5. Metabolic Management and Nutritional Complications

Metabolic complications associated with nutritional therapy remain clinically relevant. Borriello et al. (Contribution 6) provided a comprehensive review of refeeding syndrome in critically ill patients, emphasizing the importance of early identification and prevention in high-risk populations.

Adams et al. (Contribution 7) evaluated different neutral protamine Hagedorn (NPH) insulin dosing intervals in hyperglycemic trauma patients receiving continuous enteral nutrition. Although glycemic control outcomes were similar between dosing strategies, their study highlights the close interaction between nutritional delivery and glucose management.

## 6. Emerging Topics in Critical Care Nutrition

Several contributions in this Special Issue explored emerging topics that may shape future nutritional practice.

Zarantonello et al. (Contribution 8) reviewed the potential role of plant-based proteins in acute kidney injury. Beyond serving as nutritional substrates, plant-derived proteins may influence oxidative stress, inflammatory pathways, and renal hemodynamics. While clinical evidence remains limited, this review introduces a novel perspective regarding protein quality and metabolic effects in critically ill patients.

Boccardi et al. (Contribution 9) examined immunonutrition in elderly acute care patients and reported shorter hospital stays together with favorable immunological responses. Although the study population was relatively small and not limited to ICU patients, the findings support continued interest in immune-modulating nutritional interventions and their potential role in selected high-risk populations.

These studies expand the scope of critical care nutrition beyond traditional nutrient delivery and suggest potential avenues for future translational research.

## 7. Future Directions

Despite substantial advances in recent years, several important questions remain unresolved. The optimal quantity, timing, and composition of protein administration during different phases of critical illness remain controversial. Although personalized nutrition is increasingly advocated, practical strategies for implementing individualized nutritional prescriptions remain underdeveloped.

Future studies should move beyond traditional outcomes such as mortality and length of stay and focus more on skeletal muscle mass, physical function, frailty, and long-term quality of life. In addition, integrating metabolic monitoring, body composition assessment, artificial intelligence-based decision support systems, and biomarker-guided interventions may enable a new era of precision nutritional therapy.

Large-scale randomized controlled trials will be essential to determine whether these evolving approaches translate into meaningful improvements in patient-centered outcomes.

## 8. Conclusions

The studies included in this Special Issue collectively reflect the evolving paradigm of critical care nutrition. Traditional nutritional support strategies primarily focused on delivering adequate calories and preventing malnutrition. Contemporary nutritional therapy increasingly seeks to optimize metabolic responses, preserve muscle mass, support multidisciplinary rehabilitation, and improve long-term recovery.

In conclusion, critical care nutrition is transitioning from conventional nutritional support toward personalized nutritional therapy. Beyond ensuring adequate nutrient delivery, modern nutritional management aims to provide the right nutrients to the right patient at the right time, preserve muscle mass, facilitate recovery, and improve long-term patient-centered outcomes. The contributions included in this Special Issue provide valuable insights into current practice and identify important directions for future research in critically ill patients.
